# A novel truncated variant in *SPAST* results in spastin accumulation and defects in microtubule dynamics

**DOI:** 10.1186/s12920-023-01759-6

**Published:** 2023-12-08

**Authors:** Jie Wang, Yihan Wu, Hong Dong, Yunpeng Ji, Lichun Zhang, Yaxian Liu, Yueshi Liu, Xin Gao, Yueqi Jia, Xiaohua Wang

**Affiliations:** 1 Department of Genetics, Inner Mongolia Maternity and Child Health Care Hospital, Hohhot, 010020 China; 2 State Key Laboratory of Reproductive Regulation and Breeding of Grassland Livestock (RRBGL), Inner Mongolia University, Hohhot, 010070 China; 3 Department of Family Medicine, Inner Mongolia People’s Hospital, Hohhot, 010057 China; 4 Department of Pediatrics, Inner Mongolia Maternity and Child Health Care Hospital, Hohhot, 010020 China

**Keywords:** *SPAST*, Hereditary spastic paraplegias, Spastin, Intracellular accumulation

## Abstract

**Objective:**

Haploinsufficiency is widely accepted as the pathogenic mechanism of hereditary spastic paraplegias type 4 (SPG4). However, there are some cases that cannot be explained by reduced function of the spastin protein encoded by *SPAST*. The aim of this study was to identify the causative variant of SPG4 in a large Chinese family and explore its pathological mechanism.

**Materials and methods:**

A five-generation family with 49 members including nine affected (4 males and 5 females) and 40 unaffected individuals in Mongolian nationality was recruited. Whole exome sequencing was employed to investigate the genetic etiology. Western blotting and immunofluorescence were used to analyze the effects of the mutant proteins in vitro.

**Results:**

A novel frameshift variant NM_014946.4: c.483_484delinsC (p.Val162Leufs*2) was identified in *SPAST* from a pedigree with SPG4. The variant segregated with the disease in the family and thus determined as the disease-causing variant. The c.483_484delinsC variant produced two truncated mutants (mutant M1 and M87 isoforms). They accumulated to a higher level and presented increased stability than their wild-type counterparts and may lost the microtubule severing activity.

**Conclusion:**

*SPAST* mutations leading to premature stop codons do not always act through haploinsufficiency. The potential toxicity to the corticospinal tract caused by the intracellular accumulation of truncated spastin should be considered as the pathological mechanism of SPG4.

**Supplementary Information:**

The online version contains supplementary material available at 10.1186/s12920-023-01759-6.

## Introduction

The hereditary spastic paraplegias (HSP) are a group of clinically and genetically diverse neurodegenerative disorders characterized by spasticity and weakness of lower limbs. To date, over more than 80 mutated genes have been defined in HSP patients [1]. HSP is classified by mode of inheritance including autosomal dominant, autosomal recessive, and X-linked traits. Autosomal-dominant hereditary spastic paraplegia-4 (SPG4, OMIM #182,601) is the most common form, counting to around 45% of cases [2, 3]. The prevalence of SPG4 is estimated to 1–5/100,000 worldwide [4].

SPG4 is caused by the heterozygous pathogenic variants in the *SPAST* gene (MIM 604,277) on chromosome 2p22. Spastin, encoded by *SPAST*, belongs to the ATPases associated with diverse cellular activities family of proteins. Spastin is a microtubule severing protein that breaks longer microtubules into shorter ones [5, 6]. Errico et al. showed that spastin interacts with microtubules. Interaction with the cytoskeleton was mediated by the N-terminal region of spastin and was regulated through the ATPase activity of the AAA domain [5].

Spastin has two major isoforms including a full-length isoform called M1 (68 kDa, 616 amino acids), and N-terminal truncated (1–86 amino acid) short isoform called M87 (60 kDa, 530 amino acids) [7]. Full length spastin consists of functional domains including the hydrophobic domain (HD) (residues 1–87), the microtubule interacting and trafficking domain (MIT) (residues 116–194), the microtubule-binding domain (MTBD) (residues 270–328) and the adenosine triphosphatases associated with diverse cellular activities (AAA) domain (residues 342–599) [8]. The MIT, MTBD and AAA domains are present in both M1 and M87 spastin isoforms, whereas the 86-amino acid N-terminal domain is present only in M1 spastin. M1 is mainly distributed in the adult spinal cord, whereas M87 is more abundant and ubiquitously expressed than M1 [9]. In the subcellular localization, M1 spastin particularly localizes to the endoplasmic reticulum membrane (ER) and lipid droplets (LD) because the hydrophobic N-terminal domain plays a key role in wedging into the ER and LD membrane. In comparison, M87 distributed in the whole cell [7, 10].

To date, the disease mechanism is not adequately understood. Loss of function resulting from inactivating mutations in one *SPAST* allele is the most postulated explanation [11]. However, there is a growing evidence that decreased severing of microtubules does not fully explain HSP-SPG4 [12, 13]. Recently, Chen et al. reported that cytotoxicity caused by intracellular accumulation of mutant spastin may be the explanation [14].

In this study, we recruited a large pedigree with family history of SPG4. Whole exome sequencing (WES) identified a novel null variant (NM_014946.4:c.483_484delinsC) in *SPAST*. The variant produced two truncated spastin isoforms lacking the microtubule severing activity. More important, the stability of mutant proteins was increased significantly than the wild-type counterparts. Together, the potential toxicity to the corticospinal tract caused by the intracellular accumulation of truncated spastin should be considered as the pathological mechanism of SPG4.

## Materials and methods

### Patients and ethnic approval

A five-generation family with autosomal dominant HSPs were identified from the Inner Mongolia Autonomous Region, China. Magnetic resonance imaging scans of the brain and spinal cord, neurophysiological examinations including sensory nerve conduction studies, motor nerve conduction studies combined with F-ware analysis, and somatosensory evoked potential studies were performed. Disease severity was assessed using the Spastic Paraplegia Rating Scale [15].

Written informed consent was obtained from the participants or legal guardians. The study was approved by the Ethics Review Committee of Inner Mongolia Maternity and Child Health Care Hospital (approval number: 2020-018-1) according to the Declaration of Helsinki.

### Genetic analysis

**Fig. 1:**
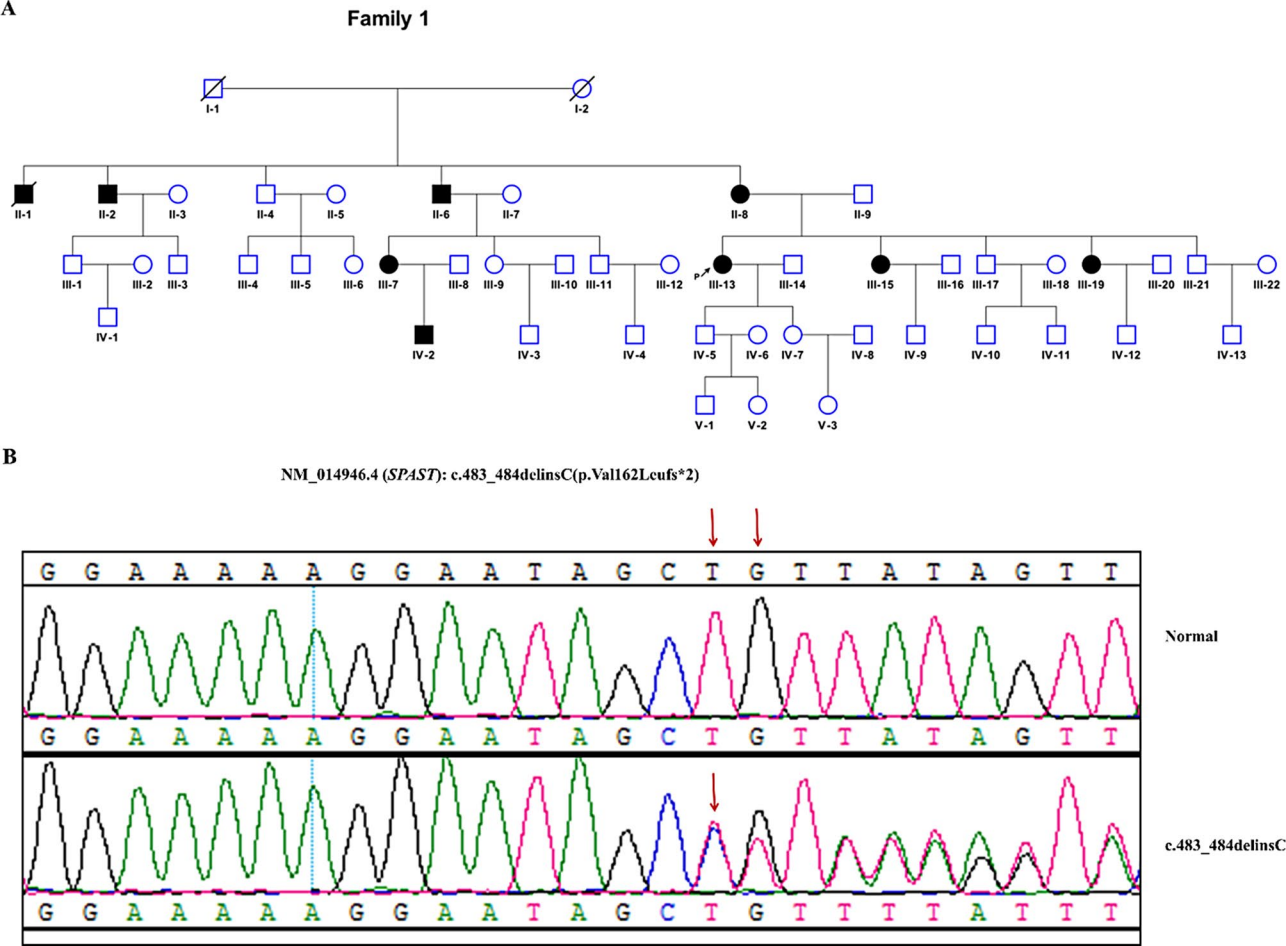
Pedigrees of the hereditary spastic paraparesis (HSP)-affected family. (**A**) the pedigree. (**B**) Sanger sequencing confirms the presence of NM_014946.4:c.483_484delinsC variant in spastin cDNAs in II-2, II-6, II-8, III-7, III-13, III-15, III-19. A normal genotype was confirmed in II-4, II-9, III-17, III-21, IV-5, IV-7. The pedigree suggests an autosomal dominant inheritance. HSP-affected individuals are marked by filled black symbols; individuals with unclear disease status are marked by filled gray symbols; the proband is marked with an arrow

Peripheral blood (10 mL) was collected. Genomic DNA was extracted using a QIAamp DNA Mini Kit (Qiagen, Germany). WES was carried out for the patient (Fig. 1, II-2、II-6、II-8、III-7、III-13、III-15、III-19) and (II-4). Exome was captured using Roche KAPA HyperExome probes according to the manufacturer’s instructions (Roche Diagnostics, Indianapolis, IN, USA). Specifically, the library was constructed and sequenced at paired-end 100 on an MGISEQ-2000 sequencer (BGI Genomics, Shenzhen, China). The sequencing data were aligned to the human reference genome (hg19/GRCh37) using BWA [16]. GATK [17]. The Ensembl Variant Effect Predictor were employed for variant calling and variant annotation [18]. The allele frequency of each variant was aggregated from the gnomAD database [19]. Common single nucleotide polymorphisms, defined as a minor allele frequency > 0.1%, were filtered. Finally, variants were interpreted based on the variant interpretation guidelines recommended by the American College of Medical Genetics and Genomics and the Association for Molecular Pathology (ACMG/AMP) [20]. The reported variant was confirmed by Sanger sequencing (Supplementary Table 1).

### Real time quantitative PCR

Total RNA was extracted from Peripheral blood and HEK293T cells using a Trizol kit according to manufacturer’s instructions. Total RNA was subjected to reverse transcription using the cDNA synthesis kit (YEASEN Biotech, China). Real time quantitative PCR (RT-qPCR) was carried out using SYBR green PCR master Mix (Toyobo, Japan). mRNA levels were normalized to *GAPDH*. The primers were listed in Supplementary Table 3.

### Plasmid construction

To investigate the cellular localization of mutant spastin, we constructed four clones into the pEGFP-C1 expression vector. Specifically, the full-length *SPAST* cDNA sequence was cloned into the vector for the expression of wild-type M1 isoform. The wild-type M87 plasmid was obtained by deleting the N-terminal part encoding the first 86 amino acids. The mutant expressing plasmid (MUT-M1 and MUT-M87) was generated using site-directed mutagenesis (Sangon Biotech, B639281). The plasmids were confirmed by sequencing; the primer sequences are shown in Supplementary Table 2.

### Cell culture and transfection

HEK293 cells were cultured in DMEM cell culture medium containing 10% fetal bovine serum (Gibco, Grand Island, NY, USA) and incubated at 37℃ in 5% CO_2_ (SANYO). Transient transfection was performed using Lipofectamine 3000 (Invitrogen, CA, USA). 3 × 10^5^ HEK293 cells were seeded in a 6-well plate. The transfection efficiency was evaluated by counting the number of spastin-positive cells per 200 cells. After transfection, the culture plate was placed in a CO_2_ incubator at 37 °C for 48 h for subsequent experiments.

### Isolation of protein lysate

Cultured cells were subjected to several rounds of washing using 5 mL of cold PBS to remove culture medium. Trypsin digestion solution was added to the cells, followed by incubation at 37 °C for digestion. After digestion, the cells and digestion solution were transferred to a centrifuge tube and centrifuged at 1000 rpm for 10 min. The pellet was washed twice with cold PBS using a centrifugation at 1000 rpm for 5 min each time. For every 1 mL of cold lysis buffer, 10 µL of phosphatase inhibitor, 1 µL of protease inhibitor, and 5 µL of 100 mM PMSF were added and mixed well. This mixture was kept on ice for several minutes. After washing the cells, they were transferred to a new pre-cooled 1.5 mL centrifuge tube. The previously prepared cold lysis buffer was added to the tube. The mixture was placed in an ice bath and incubated for 30 min, and then was centrifuged at 12,000 rpm and 4 °C for 15 min. The resulting supernatant contained the protein lysate.

### Protein stability analysis

The cells were transfected with 1.5 µg plasmid, cultured for 48 h and then treated with 500 µM cycloheximide (Cycloheximide; MCE, China) for 0, 4, 8, and 12 h, respectively. The accumulated levels of wild-type and mutant spastin isoforms were detected by Western blot analysis. All experiments were performed three times independently.

### Immunofluorescence

After transfecting HEK293 cells and culturing for 24 h, cells were fixed with 4% formaldehyde for 30 min, and then permeabilized with 3% H_2_O_2_-methanol solution. Blocking with goat serum for 30 min, the tubulin was detected by anti-tubulin antibody (Rabbit anti-tubulin, 1:50, 7634 S, CST, USA) for 37℃ in 2 h and then incubated with a secondary antibody (Goat-anti-TRITC, 1:100, KGAA99, KeyGen Biotech, China) for 37℃ in 1 h. The nuclei were stained with 4,6-diamidino-2-phenylindole (DAPI). Images were captured using an FV1000 confocal microscope (Olympus, Tokyo, Japan).

### Western blotting

Protein samples were separated by 10% SDS-PAGE and subsequently transferred to a PVDF membrane. The membrane was then incubated with the indicated primary antibodies (anti-GFP antibody, Dia-an Biotech, anti-GAPDH, CST, anti-NPTII, Bioss) overnight at 4℃, and then reacted with Goat anti-Mouse/Rabbit IgG coupled to horseradish peroxidase.

### Statistical analysis

Significant differences were determined by Student’s t-test and one-way ANOVA. Data were considered statistically significant at *P* < 0.05 and presented as the mean ± standard error of the SEM.

## Results

### Clinical features

The pedigree is a five-generation family with 49 members including nine affected (4 males and 5 females) and 40 unaffected individuals in Mongolian nationality (Fig. 1A). Of eight affected patients, II-1 was deceased; the remaining seven was clinically diagnosed with SPG4. The average age onset 33 ± 14 (range 3–47) years old. The age onset for male and female patients was 43.5 ± 4.9 and 29.4 ± 14.7 years old, respectively. The proband (III-13) developed weakness and stiffness of lower limbs at the age of 36 years old. The severity was progressive. She needed walk aids at the age of 48, and wheelchair dependence at the age of 57. All seven patients present pure HSP with abnormalities (hypertonia, hyperreflexia, and weakness) in lower limbs. The detailed clinical features were listed in Supplementary Table 4.

### Variant analysis

**Fig. 2:**
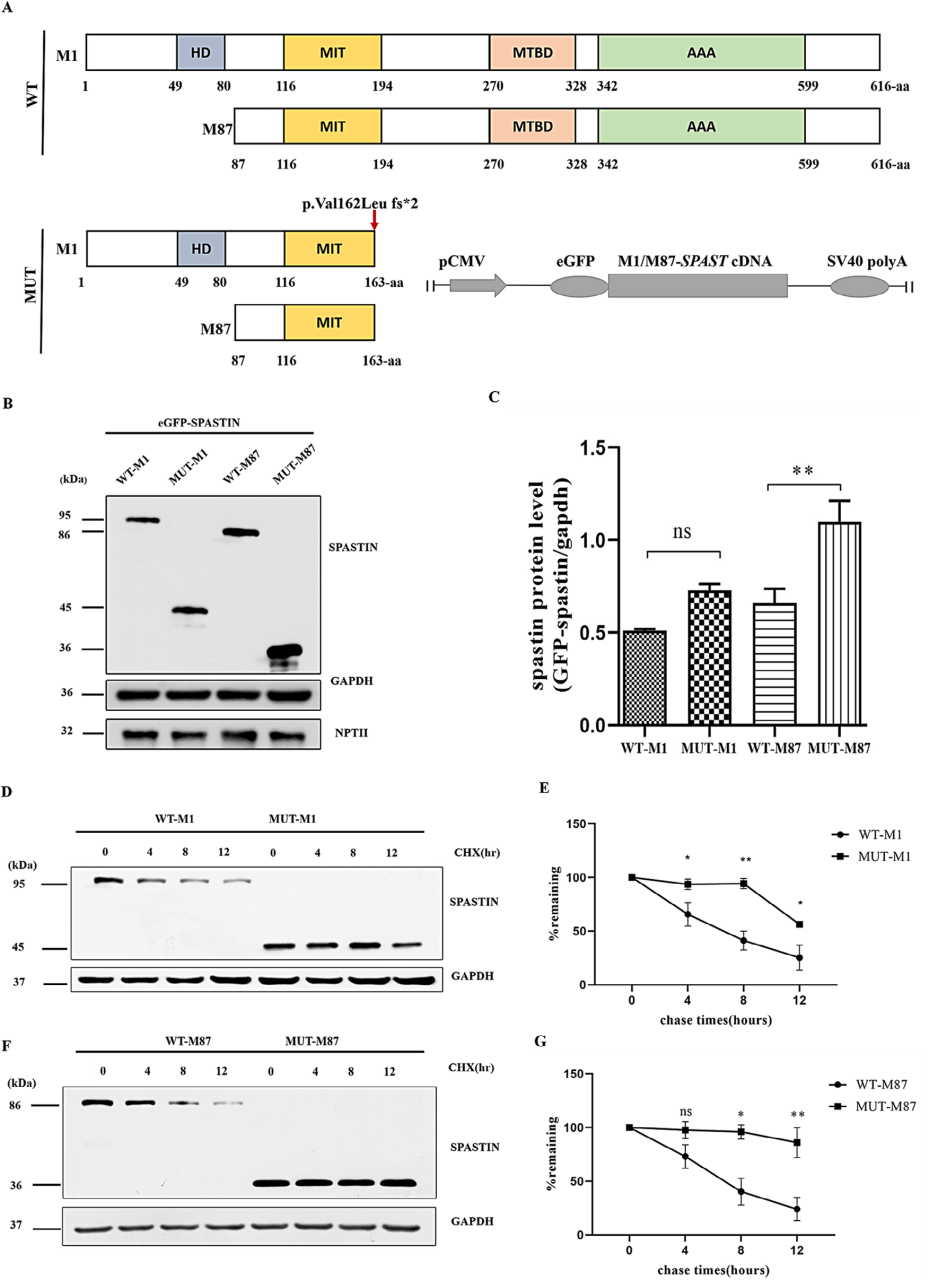
Characterization of *SPAST* c.483_484delinsC. (**A**) Schematic structure of the human wild-type (WT) and mutant spastin proteins. The red arrow indicates the location of c.483_484delinsC (p.Val162Leufs*2). M1, spastin isoforms (68 kDa); M87, spastin isoforms (60 kDa). Schematic of the spastin expression vectors is shown in the bottom right corner. (**B**) The protein expression of WT-spastin (WT-M1 and WT-M87) and c.483_484delinsC-spastin (MUT-M1 and MUT-M87). (**C**) Graphical representation of protein levels in (**B**). (**D**) Time-course stability analysis of mutant spastin (V162Lfs*2-M1). CHX, cycloheximide (500 µM). (**E**) Statistical analysis of (**D**). All values were normalized to those of untreated controls. (**F**) Time-course stability analysis of mutant spastin (V162Lfs*2-M87). (**G**) Statistical analysis of (**F**). Data represent the mean ± SEM from three independent experiments. ns, P>0.05, *P < 0.05, **P < 0.01

WES identified a novel null variant (NM_014946.4:c.483_484delinsC) in exon 2 in *SPAST*. The variant alters valine to leucine at amino acid 162, and finally generates a premature termination codon at amino acid 164 (NP_055761.2:p.Val162Leufs*2) (Fig. 1B). The frameshift variant produces truncated spastin proteins with the completely absent of the MTBD and AAA domains (Fig. 2A). The variant does not exist in gnomAD. Sanger sequencing confirms the presence of this variant in seven affected patients (II-2, II-6, II-8, III-7, III-13, III-15, III-19). A wild-type variant was confirmed in unaffected individuals including II-4, III-17, III-21, IV-5 and IV-7 (Fig. 1A). Based on the variant interpretation guideline recommended by the ACMG/AMP, the variant was classified as Pathogenic (PVS1, PM2_supporting, PP1_Strong), and was considered as the disease-causing variant in this pedigree.

### Spastin expression analysis

We first applied the minimum free energy method to predict the stability of the RNA secondary structure [21]. As a result, there was no significant changes in the mutant mRNA (-1468.10 kcal/mol vs. -1465.60 kcal/mol) (Supplementary Fig. 1A and 1B). Furthermore, an affected individual (IV-12) and two affected patients (III-15 and III19) were recruited for RNA expression analysis. The expression level of mRNA in patients’ blood did not change significantly compared with healthy control individual (Supplementary Fig. 1C), indicating that the c.483_484delinsC variant had no significant effect on RNA stability.

To answer whether the expression of N-truncated *SPAST* variants was the result of mRNA degradation, we measured *SPAST* mRNA levels by RT-qPCR using specific primers (p-3’_2_) designed to amplify a sequence close to the 3’end of *SPAST* mRNA and primers designed to amplify different sequences close to the 5’-end of *SPAST* mRNA (p-5’_1_) (Supplementary Fig. 2). Then, the ratio between mRNA levels (i.e. p-5’/p-3’) of wild-type *SPAST* and mutant *SPAST* was calculated. As a result, the normalized p-5’/p-3’ ratio of the mutants was similar to that of for wild-type in the HEK293 cell lines, indicating that no mutation-dependent degradation of 5’mRNA occurred. Thus, the expression of N-terminal truncated *SPAST* variants was enabled at the level of translation and was not regulated at the transcriptional level, nor was the result of mRNA degradation.

Given spastin has two major isoforms including a full-length isoform called M1 (68 kDa), and N-terminal truncated short isoform called M87 (60 kDa), protein expression levels of M1 and M87 were analyzed in HEK293 cells. Western blot analysis showed that the protein levels of the truncated eGFP-tagged c.483_484delinsC-M87 (MUT-M87) were higher than those of the wild-type counterpart, whereas c.483_484delinsC-M1 (MUT-M1) was higher but not reached significant difference than the wild-type counterpart (Fig. 2B and C).

To further confirm whether the stability of mutated spastin was increased, a CHX (500 µM) chase assay was performed in HEK293 cells transiently expressing eGFP-tagged wild-type (WT-M1 and WT-M87) and mutant spastin (MUT-M1 and MUT-M87) to inhibit de novo protein synthesis. The wild-type spastin protein presented rapid degradation with increased CHX treatment time from 0 to 12 h. In comparison, the two mutants (MUT-M1 and MUT-M87) did not degrade significantly (Fig. 2D-G). Together, these results confirmed the stability of mutant spastin protein was increased.

### Spastin localization and microtubule severing activity

**Fig. 3:**
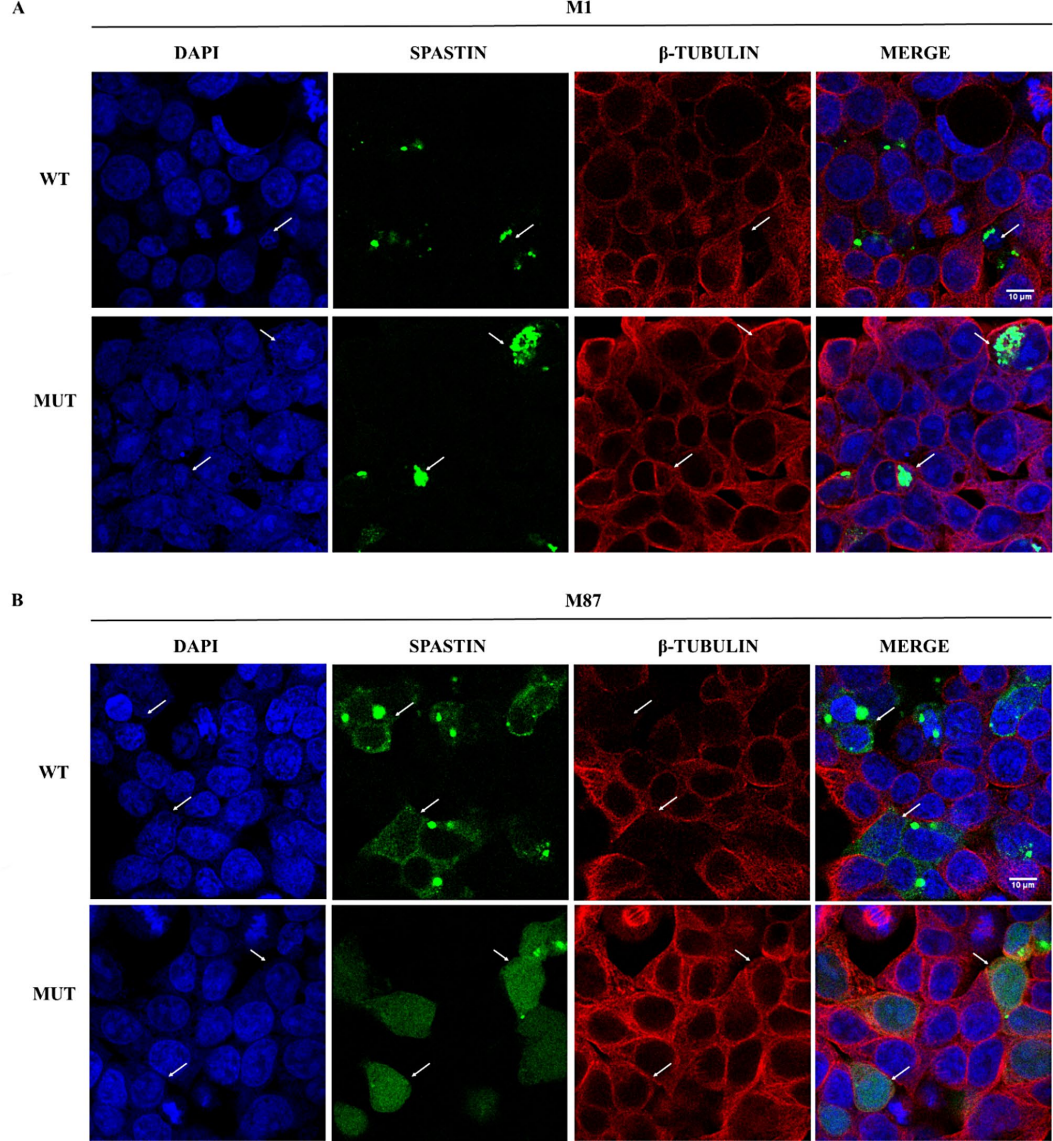
Effects of c.483_484delinsC(p.V162Lfs*2) on spastin localization and microtubule integrity in HEK293 cells. (**A**) the subcellular localization of green fluorescent protein (GFP)-tagged spastin in wild-type and mutant spastin M1 isoform, and their microtubule-severing activity. (**B**) the subcellular localization of GFP-tagged spastin in wild-type and mutant spastin M87 isoform, and their microtubule-severing activity. WT, wild-type. M1, spastin M1 isoform. M87, spastin M87 isoform. Representative immunofluorescence images for spastin (green), α-tubulin (red), and nuclei (blue) were shown

The correct localization of spatin is important for its function of microtubule severing [22, 23]. To investigate the localization of mutant spastin, eGFP-tagged wild-type (WT-M1 and WT-M87) and c.483_484delinsC-*SPAST* (MUT-M1 and MUT-M87) were transiently transfected into HEK293. As a result, the wild-type spastin showed a punctate expression pattern in cells (Fig. 3A and B), which was in line with previous studies [5, 14]. In comparison, the mutant M1 isoform was accumulated in cytoplasm, whereas the mutant M87 isoform expressed in the cytoplasm and nucleus. Staining microtubules with β-tubulin antibody, it showed that the microtubule-severing activity of MUT-M1 and MUT-M87 might be defective (Fig. 3).

## Discussion

In this study, we reported a novel frameshift variant c.483_484delinsC (p.Val162Leufs*2) in the *SPAST* gene; this pathogenic variant segregated in a five-generation family with 49 members including nine affected and 40 unaffected individuals. Patients in this pedigree presented abnormalities in low limbs and no other symptoms was observed. Together, these patients were diagnosed as apparent pure HSP. Notably, the age of onset and severity varied dramatically amongst the patients, in line with previous reports [24]. The high inter-familial variability may be explained by the presence of genetic modifiers [3].

The frameshift c.483_484delinsC (p.Val162Leufs*2) variant produced a premature stop codon and led to the synthesis of two truncated spastin isoforms (MUT-M1 and MUT-M87). Our data suggested the two mutant spastin isoforms lost the microtubule-severing activity. This could be explained by the alternations of spastin structure. Spastin M1 and M87 shares three critical domains (MIT, MTBD, and ATPase AAA domain domains) [25]. The c.483_484delinsC variant results in truncated spastin protein with the absent of MTBD domain and ATPase AAA domain (Fig. 2A). Functional analyses of truncated spastin cDNAs have revealed that these two domains are sufficient for microtubule severing [23].

Loss of function was a popular explanation of the mechanism for SPG4. The major cause of HSP-SPG4 was postulated to be insufficient microtubule cutting caused by haploinsufficiency of spastin [25, 26]. This was supported by the spectrum of mutations found in HSP-SPG4. More importantly, most of them affect spastin domains necessary for its function, inadequate microtubule severing resulting from inactivation of one spastin allele (haploinsufficiency) [25]. On the other hand, there is a growing evidence that decreased severing of microtubules did not fully contribute the explanation [12, 13]. Isoform specific toxic effects of truncated spastin may serve as an alternative pathological mechanism for SPG4 [14, 27]. Transcripts with null variants located upstream of the boundary premature stop codon are predicted to undergo degradation by nonsense-mediated RNA decay [28]. Therefore, truncated proteins are usually less stable than their full-length counterparts. However, our data demonstrated that the stability of truncated spastin isoforms increased with longer half-life than their wild-type counterparts. It is consistent with a recent study that the frameshift variant c.985dupA (p.Met329Asnfs*3) in *SPAST* increased and accumulated the mutant spastin to a higher level than their wild-type counterparts [14].

It is worth noting that two isoforms (M1 and M87) encoded by *SPAST* have different expression levels and microtubule-severing activity. M1 generally has lower expression level than M87 due to a weak Kozak sequence surrounding the M1 initiation codon. In comparison, a better Kozak sequence is present at the M87 initiation codon [25]. Moreover, the M87 isoform has higher microtubule-severing activity than the M1 isoform [13]. Although the expression level of mutated M1 is similar to the wild type, the potential subcellular toxic effect cannot be ignored. Previous studies indicated that mutated M1 isoform is neurotoxic [9, 27]. The accumulation of neurotoxic protein is a hallmark of many neurodegenerative diseases [29]. Of note, the findings in our study as well as published studies are based on in vitro experiments. Further investigations in vivo may be warranted.

In summary, we identified a novel *SPAST* frameshift variant, c.483_484delinsC, in a large Chinese family diagnosed with pure SPG4. Our study showed two truncated spastin isoforms may lack the microtubule-severing activity. More importantly, the stability of mutant proteins was increased significantly than the wild-type counterparts. The potential toxicity to the corticospinal tract caused by the intracellular accumulation of truncated spastin could be a contributing factor in the pathological process of SPG4.

### Electronic supplementary material

Below is the link to the electronic supplementary material.

Supplementary Material 1: The primers and clinical features of spastic paraplegia type 4 patients in this study

Supplementary Material 2: The original blots of Western blotting

Supplementary Material 3: The effects of SPAST c.483_484delinsC on mRNA expression in affected individuals and HEK293 cells

## Data Availability

The majority of data presented in the study are included in the article, further inquiries can be directed to the corresponding author.
