# Supplementary material for: A novel truncated variant in SPAST results in spastin accumulation and defects in microtubule dynamics
Source: BMC Med Genomics. 2023 Dec 8;16:321. doi: 10.1186/s12920-023-01759-6 (PMC10704811; doi:10.1186/s12920-023-01759-6)
Supplement: Supplementary file 1 — Supplementary Material 1: The primers and clinical features of spastic paraplegia type 4 patients in this study [file 12920_2023_1759_MOESM1_ESM.docx]

**Supplementary Table 1 Primers used in Sanger sequencing**

| Primer | Primer sequences (5’-3’) | Length (bp) |
| --- | --- | --- |
| SPAST- F | GGAGGTACCCAGCAATGAACA | 512bp |
| SPAST- R | ACAATCTTACCTTGTCCTGTAACTA |  |

**Supplementary Table 2 Primers for Real time quantitative PCR**

| Category | Primer | Primer Sequence(5’- 3’) | | Length (bp) | |
| --- | --- | --- | --- | --- | --- |
| P-5' | SPAST-F | | CGTCCGAGTCTTCCACAAAC | | 102 |
|  | SPAST-R | | TACCA TTCCACAGCTTGCTC | | 102 |
| P-3' | SPAST-F | | TGTGTGAAAGAAGAGAAGGGGA | | 103 |
|  | SPAST-R | | AGTACTCTGTCATCTCCAGCAG | | 103 |
|  | GAPDH-F | | CAAATTCCATGGCACCGTCA | | 123 |
|  | GAPDH-R | | AGCATCGCCCCACTTGATTT | | 123 |

**Supplementary Table 3 Primers for constructing the eukaryotic expression vector of *SPAST***

| Primer | Primer sequences (5’-3’) |
| --- | --- |
| M1-SalI F | GGATCCACCATGAATTCTCCGGGTGGAC |
| M1-BamHI- R | AAGCTTTTAAACAGTGGTATCTCCAAAG |
| M87-SalI- F | GGATCCACCATGGCAGCCAAGAGGAGCTC |
| M87-BamHI- R | AAGCTTTTAAACAGTGGTATCTCCAAAG |
| Mut- F | GGAAAAAGGAATAGCCTTATAGTTACAGGAC |
| Mut-R | GTCCTGTAACTATAAGGCTATTCCTTTTTCC |

M1, spastin M1 isoform. M87, spastin M87 isoform. Mut, mutant. F, forward. R, reverse.

**Supplementary Table 4 Clinical features of spastic paraplegia type 4 patients**

| No. | Sex | Age of examination (y) | Age of onset (y) | Disease duration (years) | Phenotype | Severity score | Spastic gait | Babinski sign | Hypertonia | | Hyperreflexia | | Weakness | | Additional feature |
| --- | --- | --- | --- | --- | --- | --- | --- | --- | --- | --- | --- | --- | --- | --- | --- |
|  |  |  |  |  |  |  |  |  | Upper limbs | Lower limbs | Upper limbs | Lower limbs | Upper limbs | Lower limbs |  |
| II-2 | M | 76 | 40 | 36 | Unknow | 4 | + | NA | NA | NA | NA | NA | NA | NA | NA |
| II-6 | M | 66 | 47 | 19 | Pure HSP | 4 | + | + | - | + | - | + | - | + | PC |
| II-8 | F | 81 | 36 | 45 | Pure HSP | 5 | NA | + | - | + | - | + | - | + | PC, SF, amyotrophy LL |
| III-7 | F | 47 | 3 | 44 | Pure HSP | 3 | + | + | - | + | - | + | - | + | PC |
| III-13 | F | 63 | 36 | 27 | Pure HSP | 4 | + | + | - | + | - | + | - | + | PC, SF |
| III-15 | F | 58 | 36 | 22 | Pure HSP | 4 | + | + | - | + | - | + | - | + | PC, SF |
| III-19 | F | 46 | 36 | 10 | Pure HSP | 3 | + | + | - | + | - | + | - | + | PC, SF, high myopia |

M = male. F = female. y, year. HSP, hereditary spastic paraplegias. SG = scissors gait. PC = pes cavus. SF=Sagging feet. LL = lower limbs. NA, not available.

Disease severity score, 1, No dyskinesia or only mild weakness and stiffness of lower limbs; 2, Moderate weakness and stiffness of lower limbs; 3, Unable to run, but still able to walk alone; 4, Hard to walk; 5, Full wheelchair dependence.
