# Supplementary material for: A novel truncated variant in SPAST results in spastin accumulation and defects in microtubule dynamics
Source: BMC Med Genomics. 2023 Dec 8;16:321. doi: 10.1186/s12920-023-01759-6 (PMC10704811; doi:10.1186/s12920-023-01759-6)
Supplement: Supplementary file 2 — Supplementary Material 2: The original blots of Western blotting [file 12920_2023_1759_MOESM2_ESM.pdf]

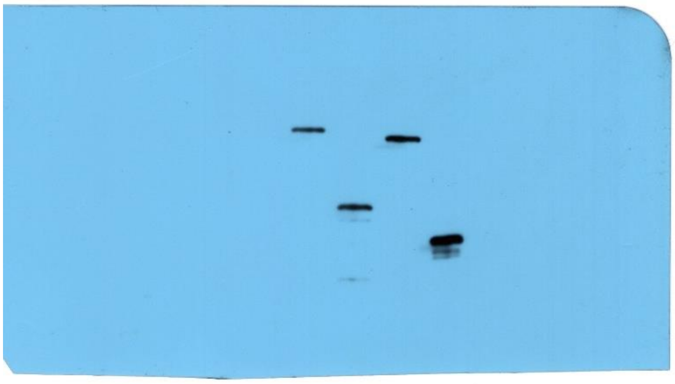

Figure original blot 1. The full length original blot of Figure2-(B)-SPASTIN.

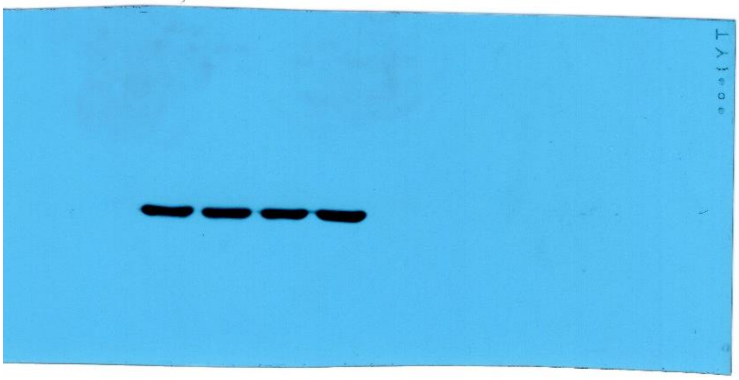

Figure original blot 2. The full length original blot of Figure2-(B)-GAPDH.

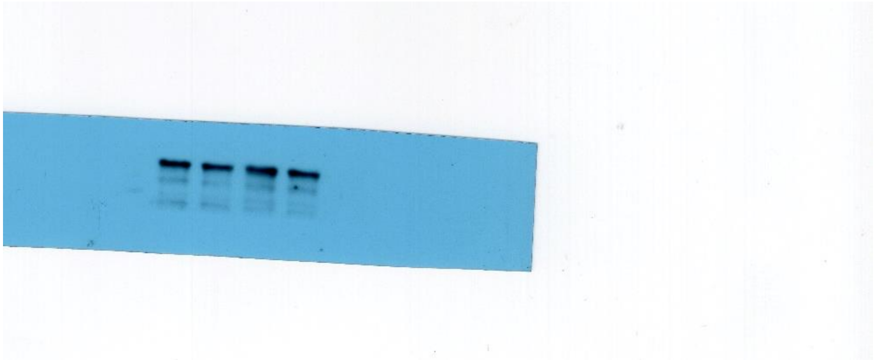

Figure original blot 3. The full length original blot of Figure2-(B)-NPTII.

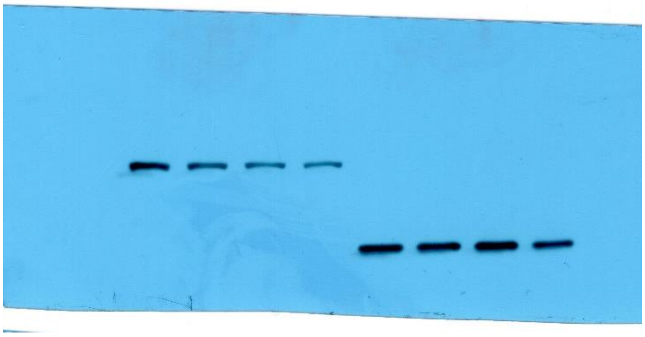

Figure original blot 4. The full length original blot of Figure2-(D)-SPASTIN-M1.

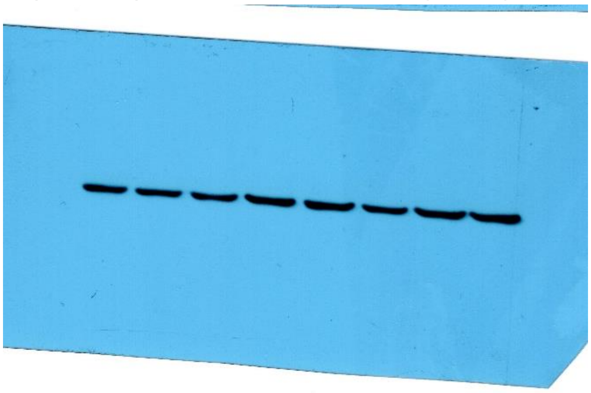

Figure original blot 5. The full length original blot of Figure2-(D)-GAPDH.

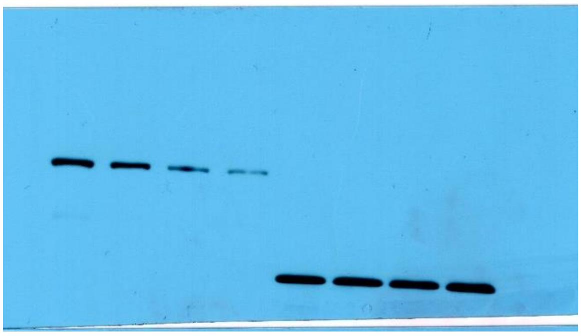

Figure original blot 6. The full length original blot of Figure2-(F)-SPASTIN-M87.

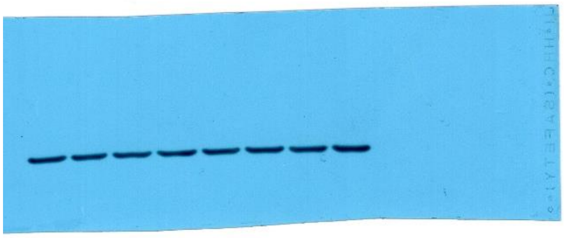

Figure original blot 7. The full length original blot of Figure2-(F)-GAPDH.
