# Supplementary material for: A novel truncated variant in SPAST results in spastin accumulation and defects in microtubule dynamics
Source: BMC Med Genomics. 2023 Dec 8;16:321. doi: 10.1186/s12920-023-01759-6 (PMC10704811; doi:10.1186/s12920-023-01759-6)
Supplement: Supplementary file 3 — Supplementary Material 3: The effects of SPAST c.483_484delinsC on mRNA expression in affected individuals and HEK293 cells [file 12920_2023_1759_MOESM3_ESM.docx]

**Figure S1. Effects of the SPAST c.483_484delinsC on mRNA expression level.** (A) MFE secondary structure of wild-type spastin mRNA. (B) MFE secondary structure of c.483_484delinsC spastin mRNA. The stability of the RNA secondary structure was predicted using the minimum free energy (MFE) method (http://rna.tbi.univie.ac.at/cgi-bin/RNAWebSuite/RNAfold.cgi). The MFE values of the WT RNA (A) and c.483_484delinsC RNA (B) secondary structures were -1468.10 kcal/mol and-1465.00kcal/mol, respectively. (C) The expression level of WT or c.483_484delinsC mRNA in patients’ blood. IV-12 is unaffected, III-15 and III19 are affected in the pedigree. No significant difference of expressive level was observed.


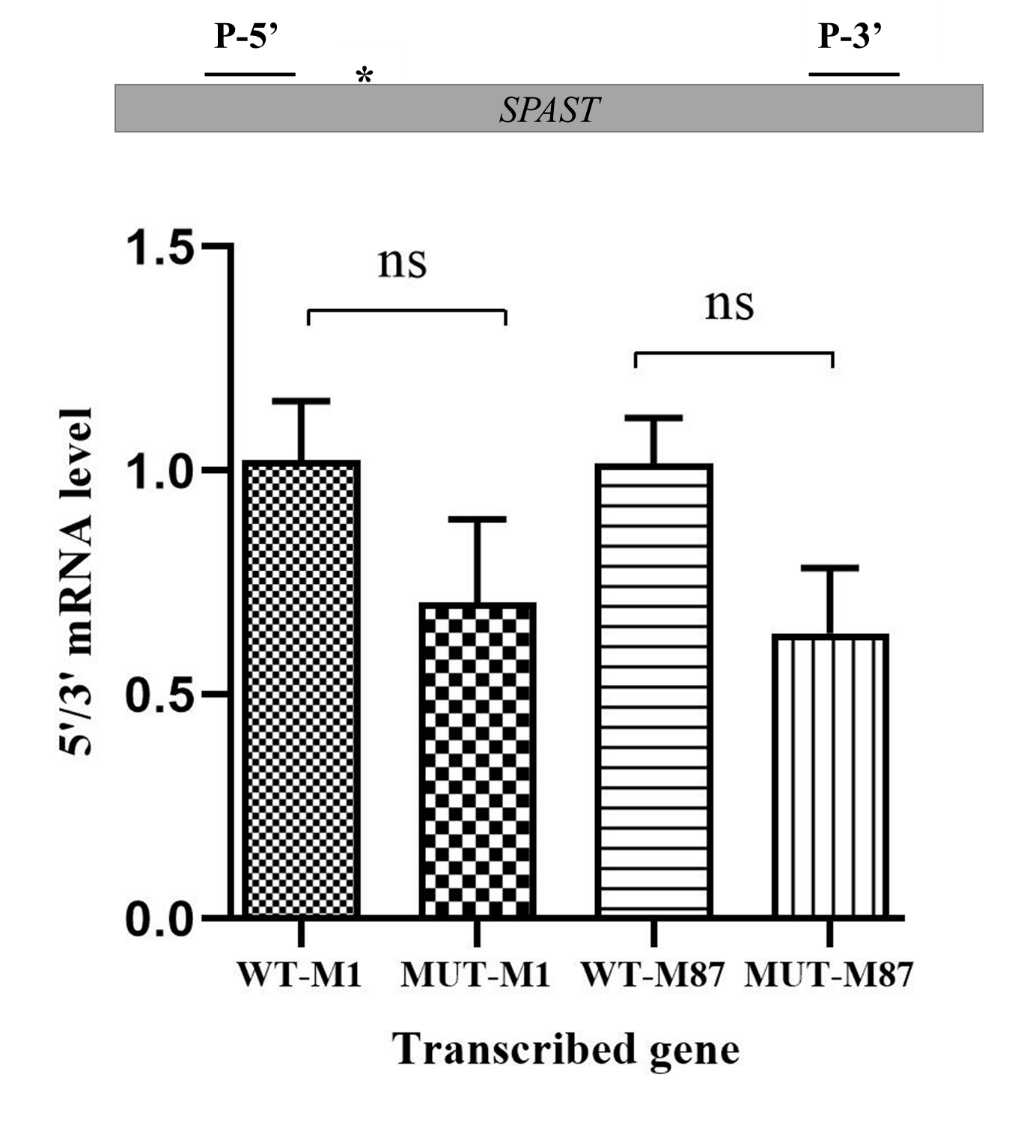


**Figure S2. The ratio between 5′ and 3′ *SPAST* mRNA levels in HEK293 cells.** mRNA levels were quantified by RT-qPCR using primers recognizing a sequence at the 5′ (p-5′) or 3′ (p-3′) end of *SPAST* mRNA. The ratios between 5′ to 3′ mRNA levels are plotted relative to those measured for wild-type *SPAST*. The asterisk indicates the truncated variant c.483_484delinsC.
